# Genetic and Chemical Modifiers of a CUG Toxicity Model in *Drosophila*

**DOI:** 10.1371/journal.pone.0001595

**Published:** 2008-02-13

**Authors:** Amparo Garcia-Lopez, Lidon Monferrer, Irma Garcia-Alcover, Marta Vicente-Crespo, M. Carmen Alvarez-Abril, Ruben D. Artero

**Affiliations:** Department of Genetics, University of Valencia, Burjasot, Spain; University of Florida, United States of America

## Abstract

Non-coding CUG repeat expansions interfere with the activity of human Muscleblind-like (MBNL) proteins contributing to myotonic dystrophy 1 (DM1). To understand this toxic RNA gain-of-function mechanism we developed a *Drosophila* model expressing 60 pure and 480 interrupted CUG repeats in the context of a non-translatable RNA. These flies reproduced aspects of the DM1 pathology, most notably nuclear accumulation of CUG transcripts, muscle degeneration, splicing misregulation, and diminished Muscleblind function *in vivo*. Reduced Muscleblind activity was evident from the sensitivity of CUG-induced phenotypes to a decrease in *muscleblind* genetic dosage and rescue by MBNL1 expression, and further supported by the co-localization of Muscleblind and CUG repeat RNA in ribonuclear foci. Targeted expression of CUG repeats to the developing eye and brain mushroom bodies was toxic leading to rough eyes and semilethality, respectively. These phenotypes were utilized to identify genetic and chemical modifiers of the CUG-induced toxicity. 15 genetic modifiers of the rough eye phenotype were isolated. These genes identify putative cellular processes unknown to be altered by CUG repeat RNA, and they include mRNA export factor Aly, apoptosis inhibitor Thread, chromatin remodelling factor Nurf-38, and extracellular matrix structural component Viking. Ten chemical compounds suppressed the semilethal phenotype. These compounds significantly improved viability of CUG expressing flies and included non-steroidal anti-inflammatory agents (ketoprofen), muscarinic, cholinergic and histamine receptor inhibitors (orphenadrine), and drugs that can affect sodium and calcium metabolism such as clenbuterol and spironolactone. These findings provide new insights into the DM1 phenotype, and suggest novel candidates for DM1 treatments.

## Introduction

Myotonic dystrophy 1 (DM1) is an autosomal dominant neuromuscular disease involving the expansion of unstable CTG repeats in the 3′ untranslated region (UTR) of the *DM protein kinase* (*DMPK*) gene. DM1 is multisystemic and characteristic features include myotonia, muscular dystrophy, iridescent cataracts, cardiac arrhythmias, and signs of neuropathology [1]. A biochemical hallmark of DM1 is misregulated alternative splicing of specific skeletal muscle, heart and brain pre-mRNAs, which explain defined DM1 symptoms such as myotonia (reviewed in [2]).

In mice, expression of 250 CUG repeats within a heterologous RNA gives rise to DM1-like phenotypes thus demonstrating that expanded CUG repeat transcripts are themselves toxic to cells [3]. Results in *Drosophila*, however, are less clear cut. Expression of 162 pure CTG repeats in the context of the 3′UTR of a Green Fluorescent Protein (GFP) reporter gene has been reported not to cause signs of pathology [4] whereas larger, interrupted, CTG repeats induced muscle degeneration [5]. Several RNA binding proteins, most notably human Muscleblind-like proteins MBNL1, MBNL2 and MBNL3, are sequestered by mutant *DMPK* transcripts. MBNL1 proteins co-localize with distinctive CUG ribonuclear foci within muscle and neuron nuclei in DM1 patients [6]–[8]. *Drosophila* model flies, though, demonstrate that ribonuclear foci are not pathogenic *per se*. RNA containing 162 CUG repeats accumulates in numerous nuclear foci together with *Drosophila* Muscleblind, but no evident pathogenic phenotype is detected [4]. DM1-associated defects are remarkably similar to those observed in *Mbnl1* knockout mice and include myotonia, ocular cataracts, histological abnormalities, and the abnormal use of specific alternative exons [9], [10]. *muscleblind* (*mbl*) loss-of-function mutations in *Drosophila* provide additional examples of DM1-like phenotypes such as missplicing of the Z-band-associated transcripts *α-actinin* and *CG30084* [11], [12].

Mbnl1 regulates a fetal to postnatal developmental switch that controls the splicing pattern of a set of murine skeletal muscle transcripts [10]. CUG-binding protein 1 (CUG-BP1) forms an RNA-dependent complex with hnRNP H that antagonizes the activity of MBNL1 proteins [13]. Both CUG-BP1 and hnRNP H are upregulated in DM1 muscle cells [13], [14] thus further contributing to the splicing pathology. Significantly, rescue experiments in DM1 model mice demonstrate that loss of *Mbnl1* function is the key event of missplicing and myotonia [15]. Additionally, overexpression of normal *DMPK* 3′UTR mRNA in mice induced up-regulation of CUG-BP1 and also reproduced cardinal features of DM1 [16].

Great effort has been put to ameliorate myotonia and abnormal cardiac conduction in DM1, which are currently treated with sodium channel inhibitors (e.g. mexiletine). Muscular weakness and wasting, or daytime somnolence, however, show little or no improvement in pharmacological trials [17]. A number of genotoxic agents suppress somatic CTG expansion mosaicism in a cell culture model [18]. PC12 neuronal cell lines expressing 250 CTG repeats exhibit cell death after cell differentiation *in vitro* that is specifically inhibited by flavonoids [19].

We previously established that *Drosophila* Mbl and human MBNL1 proteins are functional homologs [20]. Haro et al. (2006) have reported that expression of 480 interrupted CTG repeats is toxic to *Drosophila* muscle cells, that CUG RNA and human MBNL1 accumulate into ribonuclear foci, and that human MBNL1 suppresses a CUG-induced eye phenotype. Here we describe similar transgenic flies in which we confirm muscle degeneration, ribonuclear formation, and genetic interaction with *muscleblind* gene dosage. We show that CUG expressing flies reproduce additional key features of the DM1 disease including misregulated alternative splicing of muscle genes, CUG tract length dependence of phenotypes, and CUG-dependent central nervous system alterations. Furthermore, model flies were used in genetic screens and functional assays to identify new components of the pathogenesis pathway and chemical suppressors of DM1-like phenotypes, respectively.

## Results

### Continued expression of expanded CUG repeats in *Drosophila* reduces lifespan and causes muscle degeneration

**Figure 1:**
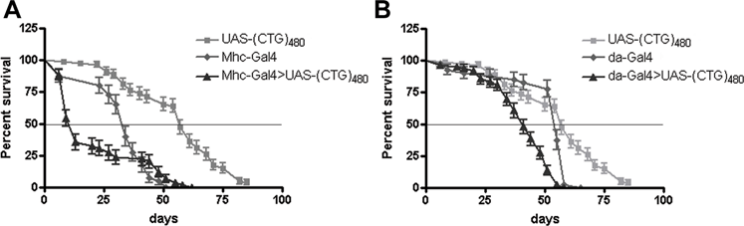
Flies expressing CUG repeats show shorter lifespan. Average percentage of live flies, with the genotypes indicated, versus age (in days). (A) Whereas control flies showed an average lifespan of 57 (*UAS-i(CTG)_480_/+*; n = 80) and 34 (*Mhc-Gal4/+*; n = 40) days, i(CUG)_480_-expressing flies lived 13 days in average (n = 60). Differences in lifespan curves were highly significant when comparing i(CUG)_480_-expressing flies to *UAS-i(CTG)_480_/+* control flies (p<0.0001, Log-Rank test) but not to *Mhc-Gal4/+* controls. (B) Expression of i(CUG)_480_ transgene in an ubiquitous manner (*da-Gal4>UAS-i(CTG)_480_*) also reduced fly survival. Control flies showed a median survival of 57 (*UAS-i(CTG)_480_/+*, n = 80) and 55 (*da-Gal4/+*, n = 40) days. Median survival for i(CUG)_480_-expressing flies was of 41 days (n = 80), and lifespan curves for i(CUG)_480_-expressing flies and both controls showed differences that were statistically significant (p<0.0001, Log-Rank test). Statistical analysis was performed using GraphPad Prism4 software.

To understand the molecular and cellular mechanisms underlying the DM1 pathology we generated transgenic *Drosophila* lines that express 60 uninterrupted or 480 interrupted CUG repeats as a non-coding transcript under the control of the Gal4/UAS system. 480 repeats consisted of synthetic CTG repeats interrupted every 20 units by the CTCGA sequence (hereafter referred to as i(CTG)_480_).The effect of expressing CUG repeat RNA in the *Drosophila* muscles or ubiquitously in the fly was studied with *Myosin heavy chain* (*Mhc*)-*Gal4* and *daughterless (da)-Gal4* lines, respectively. First we analyzed whether expression of i(CUG)_480_ RNA in *Drosophila* tissues had any impact in their lifespan. Average survival of flies expressing i(CUG)_480_ repeat RNA was lower than their corresponding control flies heterozygous for the UAS transgene or Gal4 driver. Furthermore, differences in survival curves were statistically significant except for the *Mhc-Gal4>UAS-i(CTG)_480_* and *Mhc-Gal4/+* survival curves, possibly due to a dominant effect of the *Mhc-Gal4* insertion, as this is a particularly weak stock, or the small population of flies tested (n = 40) (Figure 1). Continued expression of CUG repeat RNA in the fly musculature, or throughout the animal body, was therefore detrimental to fly survival.

**Figure 2:**
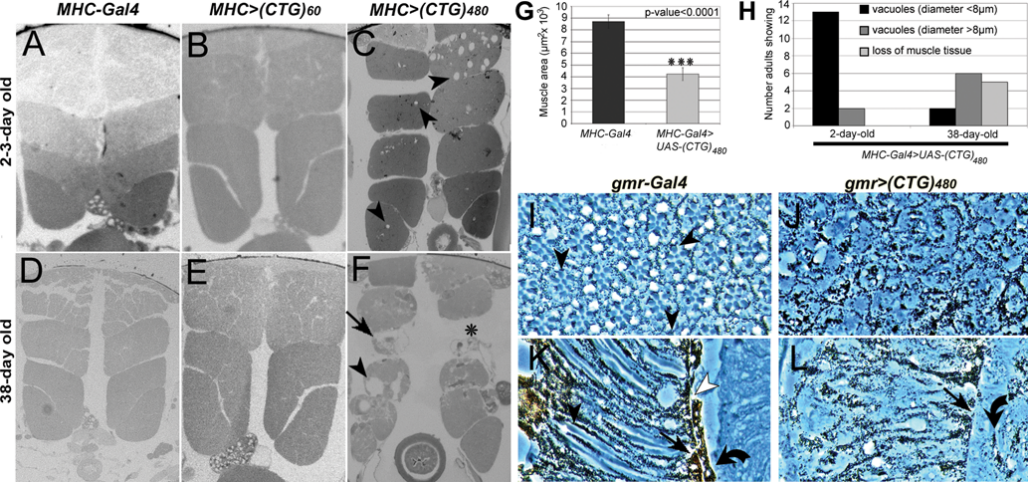
CUG-induced eye and muscle degeneration in flies. Transversal sections of resin-embedded (A–F) adult IFMs of control flies (*Mhc-Gal4/+*) (A, D) and flies expressing (CUG)_60_ (B, E) or i(CUG)_480_ RNA (C, F) under the control of the *Mhc-Gal4* driver. IFMs were studied in 2–3-day old (A–C) or 38-day old (D–F) flies. Expression of (CUG)_60_ RNA was not toxic to muscle fibres (B), and IFMs did not degenerate over time (E). Expression of i(CUG)_480_ RNA in IFMs led to vacuolization (arrowheads) and muscle disorganization (C). Muscle degeneration and wasting was conspicuous in 38-day old flies with large vacuoles (arrowheads), lower density of myofibrils per muscle (arrow) and missing muscles (asterisk). Results consistent with these have been obtained independently [5]. (G) Cross-sectional area of left dorso longitudinal muscle 45e [51] in 2 day-old control (*Mhc-Gal4/+*) and DM1 model flies (*Mhc-Gal4>UAS-i(CTG)_480_*). n = 12 (control) and n = 34 (CUG expressing). (H) Muscle degeneration was measured as the frequency of vacuolar pathology and muscle area reduction, according to the following rating scale: vacuoles with diameter larger or smaller than 8 µm, or showing 45% or less of the normal muscle area. Muscle 45e was measured in 3-4 thorax sections per animal and a total of 15 young (2-day-old) or 13 aged (38-day-old) flies were analyzed. Control (*Mhc-Gal4/+*) and *Mhc-Gal4>UAS-(CTG)_60_* flies showed no muscle phenotype 2 or 38 days after eclosion. Tangential (I, J) and frontal (K, L) sections of adult *Drosophila* eyes with the genotypes *gmr-Gal4/+* (I, K) and *gmr-Gal4/UAS-i(CUG)_480_* (J, L) at 25°C. (I) Tangential sections exhibited a normal complement of photoreceptors per ommatidial unit (arrowheads point to rhabdomeres), although some pigment cells were absent. (J) Expression of expanded CUG repeats caused general disorganization of the eye retina. (K) In controls, rhabdomeres extend from the apical to the basal side of the retina (arrowheads) and the layer of pigment cell feet forms the fenestrated membrane (arrow), which is separated by the basement membrane (white arrowhead) from the underlying subretinal cells (bent arrow). (L) Eyes expressing i(CUG)_480_ RNA lacked rhabdomeres and showed general disorganization of pigment cells. Fenestrated membranes were thinner and showed gaps (arrow). Subretinal cells were not tightly apposed to the basement membrane (bent arrow).

Flies expressing i(CUG)_480_ RNA in muscles additionally showed an age-dependent tendency to position wings upheld. These flies were flightless (n = 274) and showed alterations in indirect flight muscles (IFM), whereas those expressing (CUG)_60_ RNA did not (0% flightless, n = 204). Both *UAS-(CTG)_60_* and *UAS-i(CTG)_480_* transgenes expressed repeat RNA to similar levels (Figure S1A). 2–3 day old flies expressing i(CUG)_480_ RNA developed muscle histopathology, including vacuolization and reduction in fiber size (Figure 2A–F). We measured cross-sectional area of dorso longitudinal muscle 45e (Figure 2G). Average size of muscle 45e decreased to approximately 45% of normal when expressing 480 CUG repeat transcripts. The phenotype was degenerative as 38-day old flies had smaller IFM packages, muscles were occasionally missing, and vacuoles increased in average volume (Figure 2H). (CUG)_60_ RNA did not appreciably affect muscle organization.

Degeneration of the pigmentary retina and loss of photoreceptor neurons has been described in DM1 patients [1]. To investigate whether the retina of *Drosophila* was also susceptible to CUG repeat-mediated toxicity, we expressed i(CUG)_480_ transcripts ubiquitously in the eye-antennal imaginal disc under the control of the *glass multiple reporter* (*gmr*)-*Gal4* line. These flies showed eyes that were smaller and acutely rough. Tangential and frontal sections revealed severe alterations in the retina, including detachment of subretinal cells, thinning of fenestrated membrane and lack of photoreceptor rhabdomeres (Figure 2I–L). Expression of (CUG)_60_ RNA under the same conditions did not appreciably affect eye morphology in tissue sections (data not shown). Thus, accumulation of CUG repeat RNA in *Drosophila* muscle and eye tissue produces degenerative phenotypes that are dependent on the CUG tract length.

### CUG repeat RNA co-localizes with Muscleblind in nuclear foci

**Figure 3:**
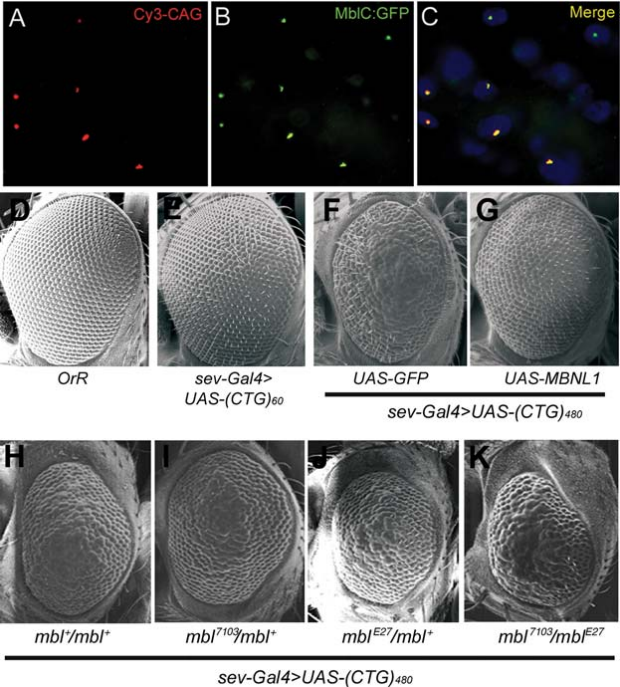
Muscleblind forms nuclear inclusions with CUG repeat RNA and genetically interacts with repeat RNA phenotypes *in vivo.* i(CUG)_480_ RNA and MblC:GFP were coexpressed in adult flies using a *hs-Gal4* line (A–C). i(CUG)_480_ transcripts detected by FISH (red; A) and MblC:GFP detected by the GFP tag (green; B). Red and green channels are shown merged in (C), with nuclei counterstained with DAPI. Scanning electron microscope (SEM) images of *Drosophila* eyes (D–K). (D) External morphology of reference strain *OrR*. (E) *sev*-*Gal4* driven expression of (CUG)_60_ RNA in eye precursors exhibits mild external defects, only altering mechanosensory bristles. Expression of i(CUG)_480_ RNA driven by the same Gal4 generates a rough and reduced eye (H), a phenotype that is specifically suppressed by the simultaneous expression of human MBNL1 (G) but not by expression of the unrelated GFP protein (F). The CUG-dependent eye phenotype was not modified by the weak *mbl^7103^* allele (I), and only slightly modified by *mbl^E27^* (J). However, the compound heterozygote enhanced roughness and eye size reduction (K). The compound heterozygote *mbl^7103^*/*mbl^E27^* displayed normal eyes in the absence of i(CUG)_480_ RNA.

Nuclear inclusions containing CUG repeats and MBNL proteins are characteristic of DM1. We investigated whether i(CUG)_480_ RNA similarly forms nuclear foci that include *Drosophila* Mbl. *mbl* encodes protein isoforms MblA, B, C and D, of which MblC has been shown to regulate alternative splicing [12]. We co-expressed i(CUG)_480_ RNA and the MblC isoform fused to the GFP (MblC:GFP) under the control of a *heat shock* (*hs*)-*Gal4* line. Simultaneous fluorescence detection in fly thorax sections showed nuclear co-localization of i(CUG)_480_ RNA and MblC (Figure 3A–C). This was not observed in controls expressing i(CUG)_480_ RNA or the fusion protein alone. (CUG)_60_ RNA did not form nuclear foci when targeted with *Mhc-Gal4* to adult musculature (data not shown). Therefore, *Drosophila* MblC incorporates into expanded CUG repeat RNA-containing foci like its human MBNL counterparts.

### *muscleblind* dose modifies CUG toxicity phenotypes

*sevenless* (*sev*)-*Gal4* driven expression of i(CUG)_480_ repeats (*sev*-*Gal4*>*UAS-i(CTG)_480_*) disorganizes ommatidia and mechanosensory bristles, and reduces eye size, which generates an externally rough eye (Figure 3H). Introduction of the weak *mbl^7103^* or strong hypomorphic *mbl^E27^* mutant alleles in this genetic background did not significantly modify eye morphology (Figure 3I, J; *mbl^E27^* may reduce size slightly). However, a clear enhancement was observed in *mbl^7103^*/*mbl^E27^* trans heterozygous flies simultaneously expressing i(CUG)_480_ RNA (Figure 3K). Conversely, targeted expression of human MBNL1 to *Drosophila* eye precursors expressing 480 interrupted CUG repeat transcripts strongly suppressed the rough eye phenotype, whereas expression of the unrelated GFP protein under the same conditions showed no effect (Figure 3F, G). 60 CUG repeat RNA caused a milder effect on external eye morphology, only altering mechanosensory bristles (Figure 3D, E). From these experiments we conclude that CUG repeat RNA compromises *mbl* function *in vivo* as similarly shown in DM1 model mice and patients [21], [15], [10].

### CUG repeat RNA induces spliceopathy in *Drosophila*

**Figure 4:**
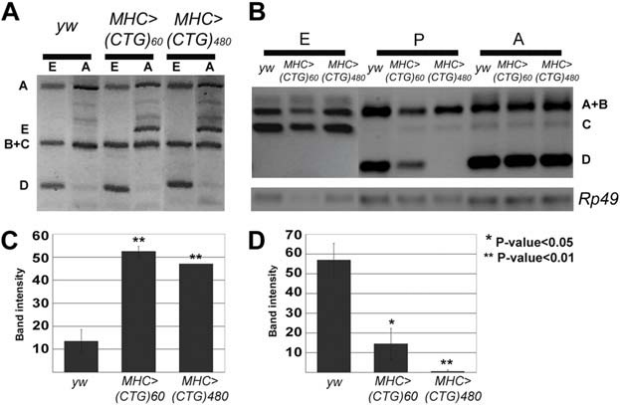
CUG repeat RNA misregulates alternative splicing of muscle genes *CG30084* and *TnT.* RT-PCR products from *CG30084* (A) and *TnT* (B) at the stages and from animals with the genotypes indicated. Bar graph representing intensities of ethidium bromide fluorescence (ranging from 0 to 100%, which equalled saturation) of band E (*CG30084*; C) and band D (*TnT;* D) from the specified genotypes. All RT-PCRs were within the linear range of amplification. Abbreviations used: 16–18 h after egg laying embryos (E); 2-day old pupae (P); 6–30 h after eclosion adults (A). All missplicing events were detected at least twice from independent RNA extractions.

Sequestration of MBNL1 correlates with missplicing events in DM1 patients. To assess whether long CUG repeat transcripts in the fly produce analogous alterations, we studied the splicing pattern of muscle genes *CG30084*, a described target of Mbl activity in embryos [11], and *Drosophila troponinT* (*TnT*) in embryos, pupae and adult flies expressing 60 CUG and 480 interrupted CUG repeat RNAs (Figure 4). Missplicing of *CG30084* pre-mRNA was conspicuous with a strong upregulation of reverse transcriptase (RT) PCR band E in adult flies expressing either 60 or 480 CUG repeat RNA (Figure 4A, C; see also Figure S2). *TnT* was similarly affected. Two-day old pupae failed to show RT-PCR band D, which was not expressed in younger pupae (data not shown), when 480 interrupted CUG transcripts were targeted to the musculature and significantly lowered its levels with 60 CUG repeat RNA (Figure 4B, D). Thus, CUG transcripts induce spliceopathy in the *Drosophila* musculature.

### Dominant genetic modifiers of a CUG-induced rough eye phenotype

**Figure 5:**
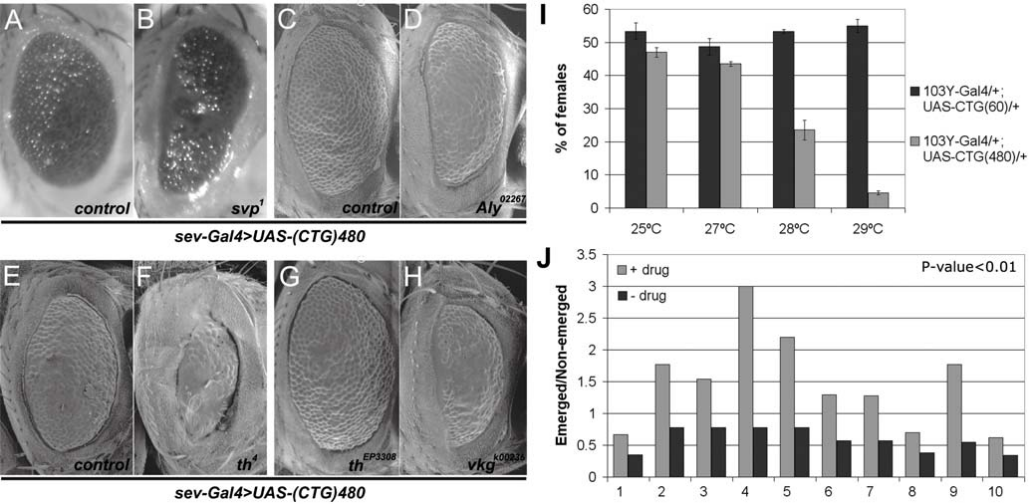
Dominant genetic enhancers and chemical suppressors of CUG-induced phenotypes. Stereomicroscope (A, B) and SEM (C–H) views of adult *Drosophila* eyes. Female flies with the genotype *sev-Gal4 UAS-i(CTG)_480_*/+ (A, C, E) show eyes smaller than normal and externally rough. Both features increased in female flies heterozygous for *svp^1^* (B), *Aly^02267^* (D), *thread^4^* (F) and *viking^k00236^* (H) in the same genetic background whereas overexpression of *th* (*th^EP3308^*, G) considerably improved morphology. Flies were raised at 25°C. (I) Percentage of viable females from crosses between the X-linked *103Y-Gal4* line and lines carrying the *UAS-i(CTG)_480_* or *UAS-(CTG)_60_* transgenes (note that only F_1_ females express CUG RNA) at different temperatures. Only expression of 480 CUG RNA exhibited a temperature-dependent semilethal phenotype. Three independent MB-specific Gal4 driver lines showed a similar behaviour. (J) Emerged/non-emerged ratio measures the likelihood of survival of CUG-expressing females in control (−drug) and drug-treated flies (+drug). Abbreviations: 1, spironolactone; 2, metoclopramide; 3, ketoprofen; 4, nefopam; 5, orphenadrine; 6, proglumide; 7, ethisterone; 8, indomethacin; 9, clenbuterol; 10, thioguanosine.

Once cardinal aspects of DM1 were confirmed in flies, we sought to identify new components of the pathogenic pathway. We performed a genetic screen of enhancer/suppressors of the *sev*-*Gal4>UAS-i(CTG)_480_* rough eye phenotype using a collection of 695 lethal P*-*element insertions and several candidate genes (Table 1, Figure 5A–H).

**Table 1:** Genetic enhancers and suppressors of a CUG-dependent rough eye phenotype

Gene	Description	Line	LOF/GOF alleles	
*cnc*	bZIP transcription factor	*l(3)j5E7*	*cnc^03921^*	S
*seven up*	orphan nuclear receptor	-	*svp^1^, svp^07842^*	E
*Nurf-38*	Nucleosome remodeling factor	*l(2)k16102*	*Nurf-38^k16102^*	S
*jumeau*	FKH/WH transcription/remodeling factor	*l(3)j8B6*	*jumu^06439^, jumu^L70^*	S
*foi*	zinc ion transporter	*l(3)j8E8*	*foi^j8E8^, foi^neo13^*	S
*viking*	alpha 2-chain type IV collagen	*l(2)k00236*	*vkg^k00236^*	E
*coro*	F-actin binding protein coronin	*l(2)k08011*	*coro^ex8^*	S
*Csk*	negative regulator of Src protein family	*l(3)j1D8*	*Csk^j1D8^*	S
*spinster*	cell death-inducing transmembrane protein	*l(2)k09905*	*spin^k09905^*	S
*thread*	inhibitor of apoptosis protein	-	*thread^4^/th^EP3308^*	E/S
*Aly*	mRNA export factor	-	*Aly^02267^*	E
*CG4589*	putative calcium binding protein	*l(2)k10502*	-	E
-	probably affects mAcR-60C or slik	*l(2)k00808*	-	S
-	unknown	*l(2)k05911*	-	S
-	unknown	*l(2)k09907*	-	E

Mutations assayed in the screen (Line). LOF/GOF column designates other loss (LOF) or gain-of-function (GOF) alleles showing interaction, or confirms that the line assayed is a known allele of the indicated gene. *svp^1^*, *thread^4^* and *Aly^02267^* were tested as candidate interacting mutations. None of the modifiers exhibit dominant eye phenotypes on their own. A second *vkg* loss-of-function allele (*vkg^01209^*) did not significantly modify eye morphology. Abbreviations: basic-leucine zipper (bZIP); fork head winged-helix (FKH/WH); Suppressor (Su); Enhancer (En).

Some modifiers are regulators of gene expression. The suppressor ca*p-n-collar* (c*nc*) encodes a bZIP protein involved in oocyte axis determination and head segment identity [22], [23]. Three Cnc protein isoforms have been described, of which CncC has been suggested to play a role in redox homeostasis [24]. We tested the ability of alleles *cnc^03921^* (disrupts all *cnc* isoforms), *cnc^EP3258^* and *cnc^EP3633^* (interrupts *cncC*) to modify the CUG toxicity phenotype. Only *cnc^03921^* dominantly suppressed the eye phenotype thus suggesting a limited or null implication of CncC in CUG toxicity. Halving the pyrophosphatase component of the Nucleosome remodelling factor (*Nurf-38*) improved eye morphology but did not suppress unrelated overexpression phenotypes in the eye (data not shown).

Additional modifiers identified genes and pathways not previously implicated in CUG-induced toxicity. Mutations in the regulators of cell adhesion and actin cytoskeleton *coronin* (*coro*) [25] and *fear of intimacy* (*foi*) [26] suppressed the phenotype. Reduction of the major structural component of basement membrane α2-chain type IV collagen (*vkg^k00236^*) enhanced the *sev*-*Gal4*>*UAS-i(CTG)_480_* phenotype.

Some modifiers of CUG toxicity control cell number. Csk negatively regulates the Src family of cytoplasmic tyrosine kinases. Mutations in *Csk*, which enlarge organs due to increased cell proliferation [27], suppressed i(CUG)_480_ RNA toxicity (*Csk^j1D8^*). Mutations in the pro- and anti-cell death genes *spinster* and *thread* were suppressors and enhancers, respectively. *Drosophila* inhibitor of apoptosis protein (Diap), encoded by the *thread* (*th*) gene, showed complex interactions. Of the three alleles tested, loss-of-function *th^4^* and *th^5^* and gain-of-function *th^6-3s^*, only *th^4^* strongly enhanced the CUG toxicity phenotype (Figure 5F). Nevertheless, *sev-Gal4* driven overexpression of *th* (*th^EP3308^*) in eyes simultaneously expressing i(CUG)_480_ significantly suppressed the phenotype (Figure 5G) whereas expression of a control GFP transgene (*UAS-GFP*) under comparable conditions did not modify eye morphology. A similar suppression was observed upon expression of the closely related Diap2 protein (*gmr-diap2* fusion construct). Furthermore, *th^4^* and *th^5^* dominantly enhanced *mblC* overexpression in the *Drosophila* eye [28].

The *sev*-*Gal4*>*UAS-i(CTG)_480_* eye phenotype was enhanced by halving the genetic dose of the mRNA export factor Aly. Several observations indicate a close relationship between mRNA export factors and exon junction complex (EJC) components [29]. However, when we tested a lethal mutation in EJC core component *tsunagi* (*tsu^EP567^*) we found no effect. In summary we identified four cellular processes likely altered by CUG repeat RNA: gene transcription, cell adhesion, programmed cell death and export of nuclear transcripts.

### Targeted expression of expanded CUG repeats to the mushroom bodies produces a temperature-sensitive pupal lethal phenotype

Mushroom bodies (MBs) are brain structures involved in learning, sleep and memory. Because of the central nervous system involvement in DM1, we targeted expression of (CUG)_60_ and i(CUG)_480_ RNA to the *Drosophila* MBs (Figure 5I). Expression with the X-linked *103Y-Gal4* driver was not deleterious at 25°C. However, an increase in the level of expression (by raising the temperature) originated a female-specific semilethal phenotype in F_1_ mature pupae expressing 480 interrupted CUG repeat RNA (Figure 5A). 28°C offered a threshold to CUG toxicity since only about 20% of emerged F_1_ individuals were females (versus 50% expected) and some died during eclosion.

Reducing the genetic dose of *mbl* in a background expressing CUG repeats in the MBs (*103Y-Gal4/+*; *mbl^E27^*/*+; UAS-i(CTG)_480_/+*) reduced the number of F_1_ females six fold compared to control flies that expressed CUG repeats only (p<0.001; Figure S3). Hence, targeted expression of i(CUG)_480_ RNA to the MBs sensitizes flies to the genetic dose of *mbl* supporting that the expression of CUG RNA in neurons reproduces a pivotal aspect of the DM1 pathogenesis, namely partial loss of *mbl* function.

### Chemical modifiers of a CUG-induced neuronal phenotype

At 28°C the semilethal phenotype of *103Y-Gal4>UAS-(CTG)_480_* flies was highly sensitive to small changes in expression of CUG RNA and was easy to quantify. It therefore provided a tool to screen chemical suppressors of the neuronal toxicity to CUG RNA. To this end, we assayed the ability of 400 compounds from the Prestwick Chemical Library (PCL), a collection of drugs selected for their biological activity, to increase viability of female flies expressing i(CUG)_480_ RNA in their MBs.

Drugs were tested individually diluted in nutritive media to ≈5 µM, which carried along the maximum amount of Dimethyl Sulfoxide (DMSO) that flies could tolerate (Figure S4), and the number of adult females was compared to controls (Figure 5J). Statistical analysis identified ten molecules (p<0.01; 2.5% of total tested) that significantly suppressed CUG-induced lethality (Table S1).

Chemical suppressors were classified into five categories according to their mechanism of action (MOA), including non-steroidal anti-inflammatory agents, and drugs showing activity on sodium and calcium metabolism (Table 2). Dopaminergic and cholinergic neurons enervate motor neurons, which are among the most abundant neuron populations in the *Drosophila* MBs [30], [31]. Two classes of compounds identified specifically acted on dopaminergic and cholinergic neurons, which suggests that i(CUG)_480_ RNA is toxic to these cell types. Genetic evidence supports this hypothesis; targeted expression of i(CUG)_480_ RNA to dopaminergic (*Ddc-Gal4*) and cholinergic (*Cha-Gal4*) neurons caused lethality (data not shown). Significantly, sodium channel blocker clenbuterol, which has been suggested effective to treat membrane excitability disorders including myotonic syndromes [32], [33], improved viability.

**Table 2:** Chemical suppressors of a CUG-induced semilethal phenotype

Biological Activity	Drug
Non-steroidal anti-inflammatory agents	*Ketoprofen* *
	*Indomethacin* *
Activity on dopamine receptors and monoamine uptake inhibitors	*Nefopam hydrochloride* **
	*Metoclopramide monohydrochloride* **
Muscarinic, cholinergic and histamine receptors inhibitors	*Orphenadrine dydrochloride* **
	*Proglumide* **
Activity on Na^+^ and Ca^+2^ metabolism	*Clenbuterol hydrochloride* **
	*Spironolactone* *
Other activities	*Thioguanosine* *
	*Ethisterone* **

* indicates p-value<0.01

** indicates p-value<<0.001

Chemical suppressors of a CUG toxicity phenotype in pupal brain. Chemical suppressors were sorted by primary pharmacological activity. Assignments are based on different online sources, mostly PubChem and DrugBank databases.

Compounds inhibiting Gal4 activity would lower transgene expression thus reducing toxicity to CUG RNA. Similarly, drugs might be working by stabilizing or degrading the CUG repeat RNA. To address these issues we first drove expression of the reporter *UAS*-*lacZ* with the *103Y-Gal4* line and measured ß-galactosidase activity in flies taking suppressor drugs and controls (Table S1). None of the chemical suppressors tested significantly altered reporter expression. Second, we measured the level of expression of 480 interrupted CUG repeat RNA under the same conditions used for the chemical screen in flies taking suppressor compounds and controls taking DMSO. Levels of expression were comparable for all tested drugs (Figure S1B). Taken together these results suggest that candidate drugs did not significantly alter expression or stability of CUG repeat RNA and thus act through alternative mechanisms.

## Discussion

*Drosophila* flies expressing 162 pure CTG repeats in the context of a 3′UTR reporter gene show no detectable pathological phenotype despite forming discrete ribonuclear foci in muscle cells [4]. This suggests that ribonuclear foci are not directly pathogenic but also that *Drosophila* might be refractory to CUG-induced toxicity since 162 pure CTG repeats are well within the pathogenic range in humans. In an attempt to express larger CTG repeat expansions, we and others [5] used synthetic, interrupted, CTG repeat minigenes [34] to model DM1 in flies. This was necessary because manipulation of large CTG repeat expansions is difficult due to their intrinsic instability and failure to amplify by PCR. Interrupted minigenes have been shown to reproduce molecular alterations characteristic of DM1, in particular missplicing of *cardiac troponin T* [34] and colocalization with Muscleblind in the cell nucleus ([35], [5]; this work). In the fly, targeted expression of 480 interrupted CTG minigenes to the eye precursors generated phenotypes sensitive to the genetic dose of *muscleblind* and in the adult musculature produced muscle degeneration ([5], this work). Furthermore, we describe missplicing of muscle transcripts (*CG30084* and *troponin T*). Although these are all alterations consistent with interrupted CTG repeats reproducing the behavior of pure CTG repeats, it remains formally possible that interrupting CTCGA repeats initiate molecular alterations unrelated to those of pure CUG repeat RNA, or somehow modify CUG-dependent toxicity. In this regard recent evidence shows that CGG trinucleotide repeats in permutation alleles of the fragile×gene (*FMR1*) cause neurodegeneration in *Drosophila* [36], [37] and involve disruption of RNA-binding protein function (hnRNP A2, Purα and CUG-BP1) as similarly described for alternative splicing regulators Muscleblind and CUG-BP1 in DM1. Thus, trinucleotide repeats similar to CTG have the capacity to cause RNA gain of function effects through mechanisms distinct from those described for CTG repeats.

DM1 was the first example of spliceopathy, i.e. expression of splice products that are developmentally inappropriate for a particular tissue. CUG repeat RNA effectively misregulated alternative splicing of Z-band component *CG30084* in *Drosophila*, leading to a strong increase of a transcript isoform we detect as RT-PCR band E (Figure 4A), whereas such isoform was almost absent in control adult flies. Similarly, expression of a *Drosophila TnT* transcript isoform we detect as RT-PCR band D (Figure 4B) was repressed in pupae expressing CUG repeat RNA, also leading to a developmentally abnormal alternative splicing. Expression of 60 CUG repeats altered alternative splicing of *CG30084* and *TnT* transcripts although these repeats did not appreciably affect muscle morphology and did not accumulate in ribonuclear foci. The apparent mismatch between molecular and cellular markers of pathology merits further consideration. First, we detect a mild eye phenotype in flies expressing 60 CUG repeats (Figure 3E) thus suggesting that 60 CTG repeats are indeed toxic to *Drosophila* cells but the phenotypes may be too weak to detect. Second, because the role of the ribonuclear foci in the disease state is currently unclear (foci are not pathogenic *per se*, at least in *Drosophila* [4]), absence of foci is not evidence that 60 CTG repeats are not toxic to *Drosophila* cells. Finally, the relevance of the alternative splicing alterations we detect in the *TnT* and *CG30084* genes is currently unknown. However, we do note that all normal alternative splicing products are detected in *CG30084* and appearance of band D is only delayed in *TnT* splicing. Therefore, we suggest that the apparent lack of match between phenotype and molecular defects in flies expressing 60 CUG repeat RNA might stem from the very different sensitivities of molecular methods and standard phenotypic assessment methods. Expectation was that flies expressing toxic RNA would show splice abnormalities typical of *mbl* loss-of-function [10]. However, we can not verify this prediction because no loss of *mbl* function phenotypes have been described in pupae and adults so far. We do notice, nevertheless, that expression of CUG repeat RNA in *Drosophila* embryos does not mimic molecular alterations described for *mbl* mutants [11], but we found inconsistencies in such description (Figure S2). It is also likely that sequestration of Mbl by CUG RNA is incomplete, thus not generating a *mbl* null-like molecular phenotype. Indeed, the splicing of *CG30084* was unaffected in *mbl* heterozygous embryos [11] demonstrating that even a reduction of 50% in Mbl protein is insufficient to interfere with splicing of *CG30084.*

Splicing of defined pre-mRNAs is defective in DM1, but the cellular readout of those changes is only beginning to be understood. The isolation of genetic enhancers and suppressors of a CUG-induced phenotype provides an unbiased approach for their identification. Our genetic screen recovered transcription and chromatin remodelling factors as modifiers. Previous observations have linked CUG toxicity to altered gene transcription [38]. Weakened cell adhesion due to impaired basement membrane, cell adhesion receptors, or both, might explain detachment of subretinal cells and sensitivity to the genetic dose of basement membrane component *vkg* and genes also influencing cell adhesion and cytoskeleton dynamics such as c*nc* [23], *coro* [25], and *foi* [26]. CUG repeat RNA might impair cell adhesion and sensitize cells to programmed cell death thus accounting for the reduction in eye size, and interaction with pro-apoptotic *spin* and apoptosis inhibitor *th*. Cell loss has been reported in specific brain areas of DM1 patients [1]. Cultured DM1 lens cells also show increased cell death, although the triggering event appears to be high intracellular Ca^2+^ levels [39]. Isolation of mutations in mRNA export factor Aly as enhancers, finally, possibly underscores the relevance of changes in nuclear accumulation of (CUG)_480_ transcripts for toxicity.

Out of 400 drugs tested we identified ten that notably alleviated neuronal toxicity to CUG RNA. Assuming that the known MOA of the suppressor drugs apply to *Drosophila,* we found a number of molecules that inhibit neuron excitation through distinct mechanisms. These include dopamine D2 receptor antagonists (metoclopramide), inhibitors of monoamine reuptake (nefopam), and muscarinic and histamine receptor blockers (orphenadrine). Mutations that decrease or increase membrane excitability are known to trigger neurodegeneration to varying degrees in *Drosophila* [40]. Expanded CUG repeats might similarly induce excitotoxicity to MB neurons. Alternatively, neuronal hyperactivation may affect motor neurons in the brain, because pupae failed to emerge but were viable if released from puparium manually.

Using our CUG RNA fly model we identified mutations and drugs that significantly modified CUG toxicity phenotypes. These results advance our understanding of the cellular processes altered by CUG RNAs and provide a proof-of-concept data that *Drosophila* DM1 models can be successfully utilized for chemical screens.

## Materials and Methods

### *Drosophila* transgenics

Construct *UAS-(CTG)_60_* was generated by subcloning 54 uninterrupted CTG repeats from the pCTG54 plasmid [41] into the *Eco*RI/*Bam*HI sites of the *Drosophila* expression vector pUAST. Sequencing of the construct revealed that repeats expanded to 60 during cloning. Because DM1 alleles carrying longer expansions probed intractable we decided to use synthetic CTG repeats interrupted every 20 CTG units by the sequence CTCGA [34]. CTG repeats in sp72 (Promega) were digested with *Xho*I and cloned into the same site in pUAST to generate the *UAS-i(CTG)_480_* construct. Both transgenes were injected into *y^1^w^1118^* embryos and independent lines established (6 *UAS-(CTG)_60_* and 14 *UAS-i(CTG)_480_*). Nine out of 14 *UAS-i(CTG)_480_* lines were crossed to *T80-Gal4*, *sev-Gal4*, *gmr-Gal4* and *Mhc-Gal4* (see below for a description of these drivers) at different temperatures of culture. Of these, seven (1.1, 2.2, 3.3, 6.4, 7.1, 9.2, 13.1) revealed externally similar phenotypes in eyes, thorax/wing positioning, or ability to fly. Subsequent experiments were carried out with transgenic line 1.1, except for the assessment of nuclear CUG repeat RNA foci formation, which was also performed with transgenic line 2.2 giving the same qualitative result. Transgenic flies *UAS-mblC:GFP* will be described elsewhere. Briefly, the coding region of *mblC* was amplified by PCR and cloned in frame with GFP into peGFP-N3 (Clontech). The entire fusion gene was excised with *Bgl*II/*Not*I and subcloned into pUAST digested with the same enzymes. Transgenic flies were generated as above.

### Fly strains and crosses

*Mhc-Gal4* was obtained from G. Davis [42]; *gmr-Gal4* from A. Ferrús (Instituto Cajal, Madrid); *sev-Gal4* from M. Mlodzik (Mount Sinai School of Medicine, New York); *103Y*-*Gal4* from J.D. Armstrong [43] and *Ddc*-*Gal4* from Mel Feany [44]. All other strains were from the Bloomington *Drosophila* Stock Center (Indiana) except for our own *mbl* mutant stocks and *UAS-MBNL1* flies [45]. *mbl^7103^* is a P{lacW} insertion approximately 57 bp upstream of the *mblA* mRNA start site [46]. *mbl^E27^* is an imprecise excision from PlacW insertion *mbl^05507b^* that removes exons 1 and 2 [47]. Females of the genotype *y^1^w^1118^; UAS-i(CTG)_480_1.1 sev-Gal4/TM3* were crossed to males from a collection of 695 *P{lacW}* lethal insertions on the 2^nd^ and 3^rd^ chromosomes [48]. To produce the genotypes shown in Figure 2A *y^1^w^1118^; UAS-i(CTG)_480_* 1.1 females were crossed to *y^1^w^1118^; hs-Gal4/CyO y^+^; UAS-mblC:GFP/+* males and adult offspring, in plastic vials, were heat shocked at 37°C for 1 h.

### Scanning electron microscopy (SEM) and histology

Adult *Drosophila* eyes and thoraces were dissected out and embedded in Epon for transversal semi-thin sectioning [49] or processed for SEM [50]. Alternatively, thoraces were embedded in OCT and transversal sections (12 µm) were taken with a Leica CM 1510S cryomicrotome. Sections were processed for *in situ* hybridization with a Cy3-labeled (CAG)_10_ probe and fluorescent detection of the MblC:GFP fusion protein as described [4]. SEM images were from a HITACHI S-2500. Image Manager Leica IM50 software was used to acquire cross-sectional muscle and vacuole areas.

### Reverse transcription-polymerase chain reaction (RT-PCR) assay

Total RNA was extracted using Tri-Reagent (Sigma). To analyze the splicing patterns, 5 µg of total RNA were treated with DNase I and reverse transcribed (RT) with SuperScriptII RNase H^−^ RT following instructions from the provider (Invitrogen). 10 µl of a 1∶25 dilution *(CG30084*), 1 µl (*Drosophila TnT*) or 1 µl of a 1:100 dilution (*Rp49*) of the RT reaction were used as template in a standard 50 µl (*CG30084)* or 20 µl (*TnT, Rp49*) PCR using TaKaRa LA Taq (*CG30084*) or *Thermus thermophilus* DNA polymerase (Netzyme, NEED) (*TnT, Rp49*) polymerases. For cycling conditions, primer sequences and annealing temperatures see supplementary materials and methods (Text S1) and Table S2.

### Compound administration and screen

Laying pots from *en masse* crosses (*yw; +; UAS-i(CTG)_480_1.1*×*103Y-Gal4/Y; +; +*) were periodically checked for first instar larvae. Ten male larvae of the genotype *yw/Y; +; UAS-i(CTG)_480_/+* and 20 female larvae with the genotype *yw/103Y-Gal4; +; UAS-i(CTG)_480_/+* were hand-picked and transferred to vials with 1 ml of Instant *Drosophila* Medium (SIGMA) containing 5 µM of compound or 0.1% DMSO in controls. 400 compounds of the PCL (Tables S3 and S4) were individually tested in triplicate. Cultures were grown at 28°C and the sex of adults scored. Males were used as internal controls to discard unviable cultures or toxic drugs. Compounds showing activity in the initial screen (p<0.01; 30 drugs) were independently tested two more times in triplicate as above. For ß-galactosidase activity readings and DMSO toxicity assays see supporting materials and methods.

### Statistical analysis

For the chemical screen, the following modification of the Fisher's exact test (α = 0.01) was used to analyze data from small size samples:
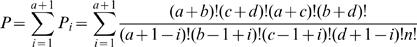
where a is emerged females from control; b is dead females from control; c is emerged females form drug treated culture; d is dead females from drug treated culture. The number of emerged and dead females after drug administration was compared to that in control cultures. Data from all replicates was summed up and treated all together in order to increase the power of the test giving a final n = 60 (initial screen), or 180 for those drugs that were re-tested. We note that because the test we developed is exact, meaning by that we know the probability of the first species error and the potency of the test, the number of false positives does not increase with the continued use of the test. A t-student test was applied to all other comparisons between two groups.

## Supporting Information

Text S1 It contains supplementary material and methods and supplementary reference list
(0.04 MB DOC)

Figure S1 Levels of transgene expression. RT-PCR detection of CUG repeat RNA from UAS-(CTG)60 and UAS-i(CTG)480 transgenes (A) and from UAS-i(CTG)480 (B), driven by the indicated Gal4 line, in the presence of DMSO (control, 1), spirolonactone (2), clenbuterol (3), metoclopramide (4), ethisterone (5), orphenadrine (6), thioguanosine (7), and ketoprofen (8) at the same concentrations used in the chemical screen. RNA from yw flies was used as negative control and Rp49 transcripts were amplified as control of input RNA. (A) Levels of expression from both the UAS-(CTG)60 and UAS-i(CTG)480 transgenes were equivalent both in pupae (P) and adult flies (A).
(4.24 MB TIF)

Figure S2 Genomic organization of CG30084 and alternative splicing isoforms detected. Exon usage in RT-PCR bands A to D (embryonic) and E (adult) according to the nomenclature used in Figure 3A. Bands A to D correspond to bands a to d in [11]. According to our results exon 12b is not detected in any RT-PCR product and exon composition of all bands shows inconsistencies with the published description [11].
(0.37 MB TIF)

Figure S3 CUG repeat RNA interferes with Muscleblind function in the brain structures the mushroom bodies. Expression of expanded CUG repeat RNA in the mushroom bodies of female flies is detrimental as only 32 individuals out of 214 were female in contrast with the expected 107 (second column; O/E ratio of 0.3). Flies heterozygous for mbl mutant allele mblE27 that simultaneously express CUG repeat RNA in their MBs show a further six fold reduction in viable female flies. 5 flies of the genotype of interest out of 368 were observed versus an expected number of 92 (O/E ratio of 0.05). Note that the presence of the transgene alone does not affect survival as the O/E ratio in males is still 1. These results show that muscleblind function is compromised in CUG-expressing MB neurons, thereby confirming the relevance of this phenotype to study DM1 defects in the brain. *** indicates p-value <<0.0001.
(0.72 MB TIF)

Figure S4 Toxicity of DMSO carrier. yw larvae were fed food containing increasing concentrations of DMSO and the number of individuals that reached adulthood was scored. Ten larvae were tested per replicate and up to five replicates were analyzed for each concentration. DMSO was not toxic up to 0.1% whereas concentrations of 0.15% or higher reduced viability in a dose-responsive manner when compared to controls; *** indicates p-value << 0.001. Bars represent standard deviations.
(0.04 MB TIF)

Table S1 Complete list of chemical suppressors of a CUG-dependent semilethal phenotype. Drugs are listed alphabetically along with their main known activity in human cells, effect on expression of the UAS-lacZ reporter (measured by the enzymatic activity of β-galactosidase), and chemical structure. β-galactosidase activity comparisons between drug-treated flies and controls were only performed when total protein quantifications found no significant differences between samples.
(0.08 MB DOC)

Table S2 Names, sequences and annealing temperatures of primers used in this work.
(0.04 MB DOC)

Table S3 Complete listing of drugs assayed in this study
(0.04 MB XLS)

Table S4 Primary data from the chemical screen. For each compound we show in columns the number of females that emerged/not emerged in drug treated and control cultures as well as the p-value of the statistical analysis. Rows contain the results from each replicate, with triplicates from independent experiments highlighted with the same colour.
(0.06 MB XLS)
