# Supplementary figures and images for: Genetic and Chemical Modifiers of a CUG Toxicity Model in Drosophila
Source: PLoS One. 2008 Feb 13;3(2):e1595. doi: 10.1371/journal.pone.0001595 (PMC2220037; doi:10.1371/journal.pone.0001595)

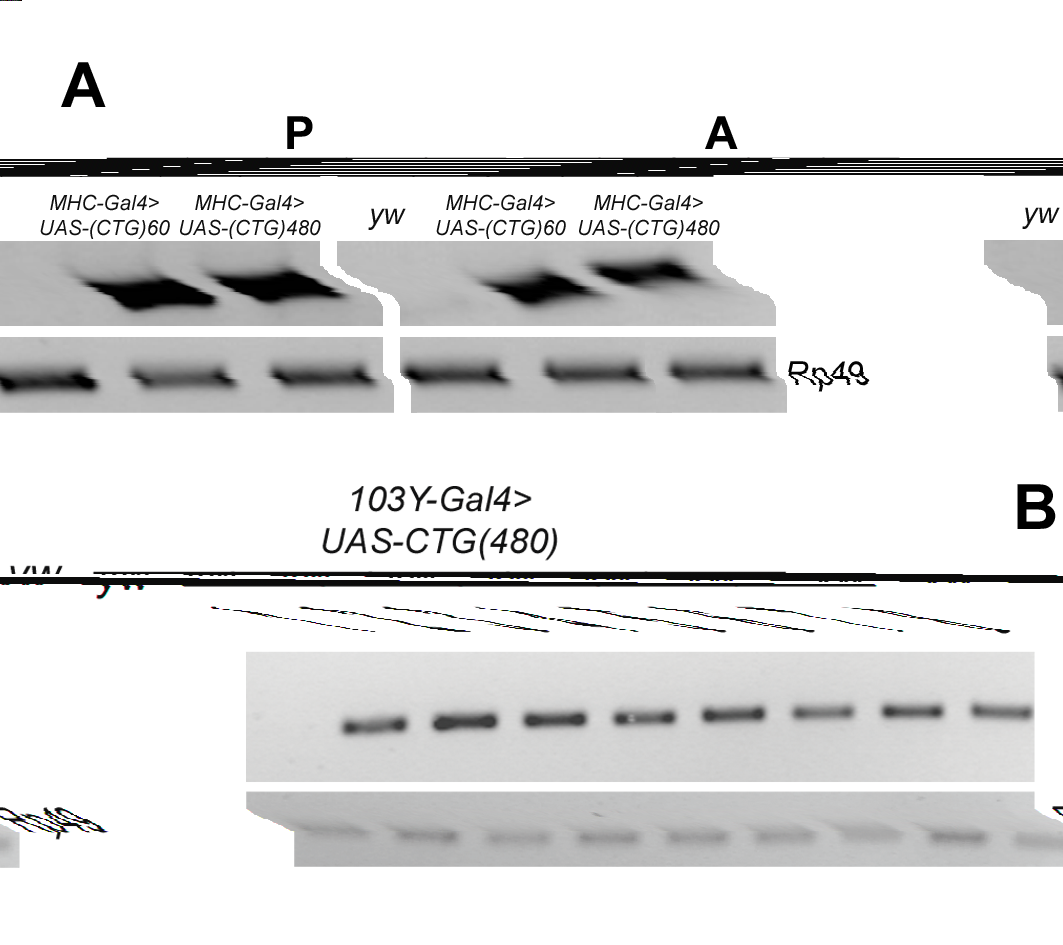

Supplement: Figure S1 — Levels of transgene expression. RT-PCR detection of CUG repeat RNA from UAS-(CTG)60 and UAS-i(CTG)480 transgenes (A) and from UAS-i(CTG)480 (B), driven by the indicated Gal4 line, in the presence of DMSO (control, 1), spirolonactone (2), clenbuterol (3), metoclopramide (4), ethisterone (5), orphenadrine (6), thioguanosine (7), and ketoprofen (8) at the same concentrations used in the chemical screen. RNA from yw flies was used as negative control and Rp49 transcripts were amplified as control of input RNA. (A) Levels of expression from both the UAS-(CTG)60 and UAS-i(CTG)480 transgenes were equivalent both in pupae (P) and adult flies (A). (4.24 MB TIF) [file pone.0001595.s002.tif]
