# Supplementary material for: Genetic and Chemical Modifiers of a CUG Toxicity Model in Drosophila
Source: PLoS One. 2008 Feb 13;3(2):e1595. doi: 10.1371/journal.pone.0001595 (PMC2220037; doi:10.1371/journal.pone.0001595)
Supplement: Supplementary file 2 [file pone.0001595.s001.doc]

Supplementary material

Supplementary materials and methods

Reverse transcription-polymerase chain reaction (RT-PCR) assay

Cycling conditions for *CG30084* were 95ºC for 1 min followed by 14 cycles of 98ºC for 15 s and 65ºC for 15 min (annealing and extension). 16 additional cycles were 98ºC for 15 s, 60ºC for 15 s and 68ºC for 15 min, and a post-PCR extension step at 72ºC for 10 min.

Cycling conditions for *troponinT* and *Rp49* were 95ºC for 5 min followed by 24 (*troponinT*) or 29 (*Rp49*) cycles of 95ºC for 30 s, 55ºC (*troponinT*) or 65ºC (*Rp49*) for 30 s and 72ºC for 1 min, and a post-PCR extension step at 72ºC for 10 min.

Detection of CUG repeat RNA

Gal4-responsive target genes are generated in *Drosophila* by cloning protein coding sequences into the pUAST vector, downstream from five Gal4-binding sites and the hsp70 TATA, and upstream of the SV40 terminator. Since the last is transcribed along with the upstream construct, and because our *UAS-i(CTG)*$_{480}$ and *UAS-(CTG)*$_{60}$ transgenes did not include sequences other than CTG repeats, we designed primers that specifically amplified a 158 bp fragment from the SV40 terminator in order to measure $(CUG)_{60}$ and $i(CUG)_{480}$ RNA levels in fly muscles (*Mhc-Gal4*) and brain (*103Y-Gal4*).

Total RNA was extracted using Tri-Reagent (Sigma). 1 μg of total RNA were treated with DNase I and reverse transcribed (RT) with SuperScriptII RNase H^- RT following instructions from the provider (Invitrogen). To amplify the SV40 terminator under the linear range of amplification, 1 µl of the RT reaction was used as template in a standard 20 µl PCR reaction with the *Thermus thermophilus* DNA polymerase (Netzyme, NEED) and specific primers SV40f and SV40r (Table S2). Cycling conditions for amplification of SV40 terminator were as for *troponinT* for 32-35 cycles. PCR product corresponding to the SV40 terminator was 158 bp long and was resolved on 2% agarose gels.

DMSO toxicity assay. 0, 0.05, 0.1, 0.15, 0.25, 0.5, 0.9 and 2.25 % of the carrier DMSO (Calbiochem) were tested for toxicity in 1 ml of food (Instant *Drosophila* Medium, SIGMA). The number of replicates ranged from 3 to 5, each containing 10 *yw/Y; +; UAS-i(CTG)*$_{480}$/+ first instar (L1) male larvae. The number of individuals that reached adulthood was compared with the number of starting L1 larvae to measure the effect of DMSO on survival. Differences were analyzed statistically with a Fisher´s exact test (α= 0.05).

Quantitative ß-galactosidase activity assay. L1 larvae from the cross *103Y-Gal4/103Y-Gal4; +; + X +/Y; +; UAS-lacZ* were fed PCL drugs or DMSO (control) as done for the chemical screen. F1 adult flies of the same sex were aliquoted into groups of three in four microcentrifuge tubes (two for each sex) and processed as described [1] with minor modifications. OD_{574} values were plotted vs time. The

slopes of the resulting lines were compared with controls using a test for equality of slopes of several regression lines (α= 0.01) to detect changes in ß-galactosidase activity. The total protein concentration of the extract was quantified as recommended in Pierce Coomassie® Protein Assay Reagent Kit, and protein levels were compared with controls with a two-tailed t-test (α= 0.05).

Supplementary reference list

1.Hazelrigg, T., 2000 GFP and other reporters, pp. 313-343 in Drosophila protocols, edited by W. Sullivan, M. Ashburner and R. S. Hawley. Cold Spring Harbor Laboratory Press, New York.
