# Supplementary material for: Genetic and Chemical Modifiers of a CUG Toxicity Model in Drosophila
Source: PLoS One. 2008 Feb 13;3(2):e1595. doi: 10.1371/journal.pone.0001595 (PMC2220037; doi:10.1371/journal.pone.0001595)
Supplement: Supplementary file 3 [file pone.0001595.s006.doc]

Table S1. Complete list of chemical suppressors of a CUG-dependent semilethal phenotype

Name	Biological activity	Effect on β-Galactosidase activity	Chemical structure
Clenbuterol hydrochloride[**]	Sodium channel blocker	none	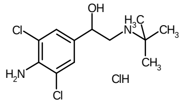
Ethisterone[**]	Antiestrogenic and antigonadotrophic activity	none	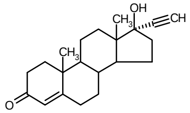
Indomethacin[*]	Cyclooxygenase inhibitor, thus blocking the synthesis of prostaglandins	not determined	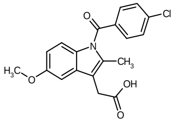
Ketoprofen[*]	Cyclooxygenase inhibitor, thus blocking the synthesis of prostaglandins	none	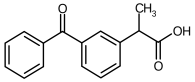
Metoclopramide monohydrochloride[**]	Dopamine D2 receptor antagonist	none	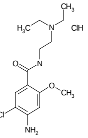
Nefopam hydrochloride[**]	Monoamine uptake inhibitor	none	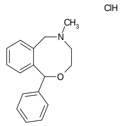
Orphenadrine hydrochloride[**]	Muscarinic receptor blocker	not determined	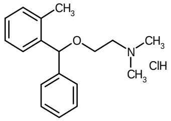
Proglumide[**]	Antagonist competitor of gastrine	none	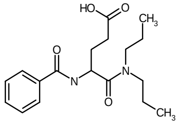

Spironolactone*	Increases amounts of sodium and water to be excreted and retains potassium	none	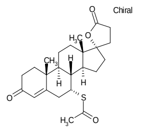
Thioguanosine*	Hypoxanthine and guanine analog, thus blocking DNA and RNA synthesis	none	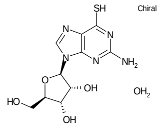

* indicates p-value<0.01, ** indicates p-value<0.001
