# Supplementary material for: Genetic and Chemical Modifiers of a CUG Toxicity Model in Drosophila
Source: PLoS One. 2008 Feb 13;3(2):e1595. doi: 10.1371/journal.pone.0001595 (PMC2220037; doi:10.1371/journal.pone.0001595)
Supplement: Supplementary file 4 [file pone.0001595.s007.doc]

CG30084 primers		
**Primer**	**Sequence (5'→ 3')**	**T annealing**
fE12a	GAACCAGGGCTATGCTCGTCC	65°C (first 14 cycles)<br>60°C (last 16 cycles)
rE16	TCCTCAACGAATCCAATGTCCTG	
fE13[1]	CAATAATAATGTGTACCGAGGTGC	
rE13[1]	GCACCTCGGTACACATTATTATTG	
fE14b[1]	TAATGTGGATAATTCCCCAGGTGC	
rE14b[1]	GCACCTGGGGAATTATCCACATTA	
fE15[1]	GGTAATCAGAATGGAGGGCAAGC	
rE15[1]	GGGTCCTGAGGTGATCTCCTTGTTG	

[1] These primers were used to sequence RT-PCR products

Rp49 primers		
**Primer**	**Sequence (5'→ 3')**	**T annealing**
fRP49	ATGACCATCCGCCCAGCATAC	65ºC
rRP49	ATGTGGCGGGTGCGCTTGTTC	65ºC

TnT primers		
**Primer**	**Sequence (5'→ 3')**	**T annealing**
fE2	CGACGATGAAGAGTACAC	55ºC
rE6	ACTCGGTGATGTATTCTTTCAG	55ºC

SV40 primers		
**Primer**	**Sequence (5'→ 3')**	**T annealing**
SV40f	GGAAAGTCCTTGGGGTCTTC	60ºC
SV40r	GGAACTGATGAATGGGAGCA	60ºC
